# The Impact of COVID-19 and Exposure to Violent Media Content on Cyber Violence Victimization Among Adolescents in South Korea: National Population-Based Study

**DOI:** 10.2196/45563

**Published:** 2024-03-22

**Authors:** Eugene Lee, Peter J Schulz, Hye Eun Lee

**Affiliations:** 1 Annenberg School for Communication and Journalism University of Southern California Los Angeles, CA United States; 2 Department of Communication, Culture and Society University of Lugano Lugano Switzerland; 3 Department of Communication & Media Ewha Womans University Seoul Republic of Korea

**Keywords:** cyber violence, adolescents, victimization, perpetration, COVID-19

## Abstract

**Background:**

Because of the COVID-19 pandemic and consequent stay-at-home mandates, adolescents faced isolation and a decline in mental health. With increased online activity during this period, concerns arose regarding exposure to violent media content and cyber victimization among adolescents. Yet, the precise influence of pandemic-related measures on experiences of cyber violence remains unclear. Hence, it is pertinent to investigate whether the pandemic altered the dynamics of cyber violence victimization for individuals.

**Objective:**

This study aims to investigate the effects of COVID-19 and exposure to violent media content on cyber violence victimization among adolescents in South Korea.

**Methods:**

We used national survey data from 2019 (n=4779) and 2020 (n=4958) to investigate the potential impact of COVID-19 on the prevalence of cyber violence among young adolescents. The data encompassed responses from elementary fourth-grade students to senior high school students, probing their exposure to violent media content, average internet use, as well as experiences of victimization and perpetration.

**Results:**

The analysis revealed a noteworthy decline in cyber victimization during 2020 compared with 2019 (B=–0.12, *t*=–3.45, *P*<.001). Furthermore, being a perpetrator significantly contributed to cyber victimization (B=0.57, *t*=48.36, *P*<.001). Additionally, younger adolescents (β=–.06, *t*=–6.09, *P*<.001), those spending more time online (β=.18, *t*=13.83, *P*<.001), and those exposed to violent media (β=.14, *t*=13.89, *P*<.001) were found to be more susceptible to victimization.

**Conclusions:**

Despite the widespread belief that cyber violence among adolescents surged during COVID-19 due to increased online activity, the study findings counter this assumption. Surprisingly, COVID-19 did not exacerbate cyber victimization; rather, it decreased it. Given the strong correlation between cyber victimization and offline victimization, our attention should be directed toward implementing real-life interventions aimed at curbing violence originating from in-person violence at school.

## Introduction

### Background

In a desperate search for meaning in the virus, many academics compared the public health dangers of the COVID-19 pandemic to the “Black Death” of the second half of the 14th century [1,2] and the influenza affliction at the end of World War I [3]. The “War,” or the Great War, as it was then called, eclipsed the number of deaths caused by the flu so that the flu, despite a tremendous number of fatalities, is not remembered as widely as the Black Death. Today’s standard living conditions are incomparable to the dire environment of medieval times. Still, violence persists in different manifestations and can severely threaten public health, thereby changing people’s sensitivity toward the pain of others. A case in point is cyber violence. Individuals inclined to harass their peers might find it easier to do so when the bully’s threshold for action is lowered. Adolescents are especially exposed to the risks of cyber violence as a result of increased online communication and learning during COVID-19.

Previous studies have shown that exposure to media violence increases the risk of cyber aggression [4]. “Cyber violence” refers to using images or videos or both in cyberspace to verbally harm or harass others [5]. Similarly, “cyber aggression” refers to behaviors that are intentionally aimed at harming others by communicating through electronic devices such as computers and mobile phones [6]. “Cyberbullying” is a form of aggression that is carried out by an individual or by a group through electronic communication consistently, over time, and with the intention of targeting a particular individual exposed to violence [7]. It is crucial to treat adolescent exposure to cyber violence seriously, especially given the heightened duration of online activities and the widespread adoption of remote learning during and in the wake of the COVID-19 pandemic. As adolescents spend more time online, they are likely to consume online content depicting violent behavior, which risks exposing them to cyber violence [8]. In this study, we examine the impact of COVID-19 on cyber violence victimization by examining survey data conducted between 2019 and 2020 among South Korean adolescents. We also examine the impact that exposure to violent media content—such as sensational content, derogatory comments attacking other people, portrayals of illegal behavior, and misinformation—has on cyber victimization.

Marciano et al [9] conducted a meta-analysis and found that adolescents around the world spent more time on digital media during COVID-19 and were more likely to experience negative effects on their mental health. Although the study did not include samples from South Korea, it illustrated the impact of COVID-19 on mental health using samples from other Asian countries (eg, China, Japan, Indonesia, and Bangladesh) and from European and Northern American countries. As exposure to media violence increases the risk of cyber aggression [4], some may argue that COVID-19 could have accelerated cyber violence. Yet, a report from Canada [10] revealed that 17% of young people actually reported a decrease in cyber victimization during the pandemic in comparison to the previous period. Similarly, American youth reported an increase in negative mental health experiences while reporting a decrease in cyber victimization during the COVID-19 pandemic [11]. Hence, due to the absence of face-to-face interactions to instigate or sustain bullying in online environments, students might have experienced a reduction in cyber victimization during the pandemic [11].

Adolescents experiencing cyber victimization are widespread across many countries. For example, 69.9% of US adolescents reported some level of being victimized by cyber harassment, with 60.8% stating that they experienced cyber harassment repeatedly [12]. According to the 2021 Cyber Violence National Survey conducted in South Korea, 30% of adolescents reported experiencing some level of cyber violence [13]. When the prolonged negative effects of cyber violence and isolation during COVID-19 converge, it can pose a significant threat to the mental health of adolescents.

Previous longitudinal studies have illustrated the prolonged negative effects of cyber violence on individuals. For instance, individuals exposed to cyberbullying continuously experience higher levels of anxiety and depression, as well as more frequent suicidal thoughts [14]. Camerini et al [14] conducted a systematic review of countries in Europe, North America, Oceania, and Asia—including South Korea—and found a prolonged negative effect of cyber violence on individuals. Hinduja and Patchin [15] demonstrated that adolescents in the United States who experienced traditional bullying or cyberbullying had more suicidal thoughts and were more likely to attempt suicide than those who had not experienced such levels of aggression.

Because of stringent government measures in response to COVID-19 in South Korea, all public facilities—including places frequented by adolescents (eg, private academies, private chat rooms, libraries, and study cafés)—were closed, thereby denying adolescents a physical space to connect with their peers during the pandemic. Byeon [16] identified experiences such as depression, subjective stress recognition, loneliness, and changes in economic status due to COVID-19 as major factors influencing suicidal ideation among Korean adolescents. Being isolated during COVID-19 has made South Korean adolescents feel disconnected from each other and from the broader community; there has also been an increase in anxiety, depression, and other indicators of psychological stress, which has increased not only the frequency of suicidal ideation but also the number of suicide attempts [17].

As adolescence marks a period of life when individuals freely consume more media due to the increased accessibility of content, adolescents constitute a significant population for studying the effects of cyber violence [18]. Engaging in activities such as surfing the internet, watching videos, social networking, and interacting with others online and offline may expose adolescents to violent content and cyber violence; this is especially likely when parental control over media consumption is limited [18,19]. More surveillance challenges arise because smartphones, unlike television or personal computers, are personalized devices that one carries throughout the day, which renders monitoring nearly impossible [20]. Collectively, these conditions and behavioral trends may explain why many adolescents have reported that they have experienced cyber violence or cyber aggression.

South Korean adolescents provide a particularly unique opportunity for researchers to study cyber violence among young adolescents. Unlike adolescents in many other parts of the world, Korean adolescents tend to possess personal mobile devices from a somewhat early age, with most starting to use smartphones during early adolescence [21]. Previous research has shown that 87.7% of elementary schoolers between fourth and sixth grades carry their mobile phones [22]. Smartphone ownership increases with age, with 92.6% of sixth graders reporting that they possess a personal smartphone. As media technology evolves, the age of owning one’s first smartphone continually decreases. Thus, the case study of Korea provides a great opportunity to prepare for a future where adolescents have more freedom and exposure to various media contents in earlier stages of their lives. In this study, we use national surveys conducted in South Korea to examine the relationship between media consumption patterns, exposure to violent content, and the experience of being a perpetrator or exposed to cyber violence.

### COVID-19 and Its Effect on Adolescents in South Korea/Asian Countries and Victimization of Cyber Violence

Previous literature centers on the mental health of Korean/Asian adolescents during COVID-19, as well as on the social impact of COVID-19 on adolescents. Mental health status and social impact, such as the level of exposure to the media, are essential factors that reflect the vulnerability of adolescents to cyber violence during COVID-19. Concerning mental health and COVID-19, studies have discussed suicidal tendencies [16,23-25], anxiety and depression [26], posttraumatic stress disorder (PTSD) [27], and the general mental health of adolescents [28,29].

Byeon [16] found that changes in economic status and deteriorated mental health during COVID-19 influenced the suicidal ideation of Korean adolescents. Similar to previous findings about isolation and suicide attempts [24], isolation during COVID-19 resulted in feelings of disconnection and increased anxiety, depression, and psychological stress among Korean adolescents, leading to increased suicidal ideation and suicide attempts [17]. Suicidal tendencies were particularly exacerbated in multicultural Korean adolescents when they were exposed to sexual intercourse, depressive moods, and unhappiness during COVID-19 [25]. Some studies have even shown that COVID-19 is associated with PTSD [27,28]. A short-term longitudinal study found that 15%-28% of the adolescents in the study showed PTSD symptoms due to COVID-19 [27].

Additionally, Field [26] reviewed several studies looking into the psychological effects of COVID-19 on adolescent mental health and found that overall, there is a significant prevalence of psychological problems—such as anxiety and depression—among adolescents in Asia and around the world. Many other psychological problems, such as stress, insomnia, social anxiety, PTSD, obsessive-compulsive disorder, and suicidality, were comorbid. These conditions were also found to be more prevalent among women than among men, and more prevalent among high school and university students compared with children. Lundström [29] reported similar findings through an analysis of 108 studies, which indicated that depression and anxiety, worrying, sleeping problems, posttraumatic stress, obsessive-compulsive disorder, deliberate self-harm, and suicidal ideation were all prevalent mental health outcomes in adolescents due to COVID-19.

The social impact category contains articles focusing on behaviors or results related to adolescents’ social life and work life [29-31]. Some studies have illustrated that COVID-19 exacerbated adolescents’ addiction to media, specifically internet addiction and game addiction. For instance, a Taiwanese study looking at 1244 junior high school students found that the prevalence rate of internet addiction during COVID-19 was as high as 24.4%. Similarly, internet game disorder became a major issue among adolescents, with a prevalence rate of 20% in South Korea during COVID-19 [30].

Some studies identified lockdowns as an important factor that led to internet or video game addiction [30]. In addition, higher levels of depression and lower levels of self-esteem, as well as subjective well-being, actual social support, and lower family function, increased the likelihood of internet addiction [31]. Similarly, previous studies have identified the relationship between internet gaming disorder and conditions of psychological well-being, such as depression, anxiety, and stress symptoms [30]. This shows that underserved adolescents with lower well-being can be more vulnerable to cyber violence during COVID-19 [32]. Most importantly, a previous study argued that increased social media use leads to cyberbullying because adolescents are more likely to be exposed to cyber violence when they spend more time online [29].

From this existing literature, we have found that COVID-19 has compromised the mental health of adolescents while increasing the time they spend consuming media, which puts them in a vulnerable situation that easily exposes them to cyber violence.

### The Relationship Between Being a Cyber Violence Perpetrator and Victimization

Cyberbullying is one of the most significant categories of cyber violence. Previous research has shown that 76% of Korean adolescents have experienced cyberbullying, and as a result, severe secondary consequences to their well-being, such as depression and possibly suicide. Cyberbullying is noted as an important aspect of examining adolescents and media. Previous studies have linked internet use [33,34], previous offline bullying, and experience of exposure to violence/bullying in school [35,36] with cyberbullying behavior. To be specific, the more adolescents use the internet, the more likely they are to be associated with cyberbullying behavior. Additionally, Hinduja and Patchin [35] argued that people who get mistreated in the real world are likely to be mistreated in cyberspace. Similarly, Ybarra and Mitchell [36] indicated that about one-half of those who are engaged in cyberbullying activities or experienced cyberbullying also experience offline bullying [36]. The literature shows no clear line between perpetrators and individuals experiencing cyber violence; being a perpetrator and victimization are correlated [37]. Further, we can say that there is no clear distinction between offline bullying and cyberbullying. In other words, offline bullying behavior can often spill over to cyberspace.

Adolescents who are exposed to cyber violence might be more susceptible to committing cyber violence toward others. As there is a correlation between perpetrators and individuals who experienced cyber violence, we include the experience of being a perpetrator in the model to explain the variance of victimization.

The quality of social interaction online seems to be an essential aspect related to well-being online. A study by Kim [38] found that for Korean adolescents, “time spent online has a deleterious effect on adolescent psychological well-being: for every one-unit increase in online social networking, the odds of suicidality rise by more than a third.” By contrast, findings from the study by Bae [39] showed that increased use of smartphones for communication results in increased social capital, which, in turn, increases adolescents’ well-being. These studies show that the types of content and kinds of people adolescents are exposed to online would have a significant impact on behaviors regarding cyber violence. If adolescents are exposed to violent content or are vulnerable interacting with others online, they are likely to get victimized. By contrast, if adolescents are having positive interactions with people online or are not exposed to violent content, it may not necessarily lead to victimization despite COVID-19 increasing their time spent online.

### The Relationship Between Violent Media Exposure, Gender, and Cyber Violence Victimization

The association between exposure to violent media content and aggressive behavior has been studied in various contexts [37,40-42]. The common premise is that people are more likely to display aggressive behaviors when exposed to violent media content. In the context of cyber violence, people exposed to violent content are more likely to be involved in cyber violence.

What about the relationship between violent media exposure and victimization? Previous studies have shown evidence of a positive correlation between being a perpetrator and being exposed to cyber violence [37,43]. In other words, individuals who commit perpetration online are also likely to experience violence, and vice versa. Therefore, we can expect a positive relationship between exposure to violent media content and victimization. However, we can also expect that people exposed to violent media content are at more risk due to the type of online community, people, and space to which they are exposed.

Gender is another crucial predictor of victimization in cyber violence. Choi and Lee [44] have examined 2 types of cyber violence: cyber harassment and cyber impersonation, and found that females are more likely to experience higher levels of victimization in cyber violence. In addition, Backe et al [45] argued that cyber violence is associated with adverse psychological and social outcomes both online and offline, disproportionately affecting women, girls, and sexual and gender minorities. By contrast, some studies have indicated that cyber offending is more associated with boys than with girls [46].

Based on our literature review, we propose the following hypotheses:

- H1: COVID-19 will affect the level of cyber victimization of adolescents.
- H2: The relationship between exposure to violent media and victimization will vary across age, gender, COVID-19, and internet usage.
- H3: Exposure to violent media content will have a positive relationship with victimization among adolescents.
- H4: Age will have a negative relationship with victimization among adolescents.

## Methods

### Overview

This study focused on exposure to violent content and cyber violence victimization before and after COVID-19. The National Survey on Cyber Violence Among Adolescents was initiated in 2013 by the Korea Communications Commission (KCC), a presidential consensus-based administrative organization. The KCC is responsible for regulating broadcasting and communication services, protecting users, and addressing related issues to maintain the independence of broadcasting services in Korea. The KCC decided to conduct the national survey in response to the serious and increasing concerns about cyberbullying as a social problem. The goal is to identify the current issues and challenges related to cyberbullying in Korea. Data from the annual survey from 2013 to 2022 will be made available upon valid request. We only selected data from 2019 and 2020 because data from other years have different items to measure the variables or incongruent response formats. The KCC delegated the 2019 and 2020 national surveys of South Korean adolescents to the National Information Society Agency, a statutory agency established in 1987 under the Ministry of Science and Information and Communications Technology and the Ministry of the Interior and Security of Korea.

### Data Collection

The data were collected among students ranging from elementary school fourth graders to seniors in high school. The academic system in South Korea has 6 years of elementary school, 3 years of middle school, and 3 years of high school. Students who start the fourth grade are usually aged 10, and third-year high school students are usually aged 18.

The data were collected using stratified random sampling. Whole classes were selected based on a regional and grade list from the School Statistics Database at the Korea Educational Development Institute. Teachers of these selected classes supervised students as they completed the survey. While students were allowed to skip individual questions, they were not allowed to skip the survey altogether. Because of this approach, the participation rate was 100%, but there are cases of missing data for certain questions.

Surveys were conducted across schools in both urban and rural South Korea, yielding 9737 responses in total (4779 participants in 2019 and 4958 participants in 2020). Participants completed the survey in schools from October 1, 2019, to November 23, 2019. However, because of the circumstances caused by COVID-19, the 2020 survey was conducted online between October 6 and November 13.

Participants comprised 5157/9737 men (52.96%) and 4580/9737 women (47.04%), and each grade had more than 1000 students participating in the study (1119, 1128, 1068, 1085, 1037, 1083, 1047, 1083, and 1087 participants from each grade starting from elementary fourth grade to third-year high school students).

### Ethics Approval

This study was granted ethical approval by the Institutional Research Board of Ewha Womans University (approval number ewha-202204-0022-02).

### Measures

A questionnaire was constructed in Korean.

### Exposure to Violent Media

Participants were asked to indicate instances in which they had been exposed to each of the 5 categories of cyber violence through various online content formats (eg, writing, cartoons, pictures, or videos) including pop-up windows. The 5 categories of cyber violence were (1) violent content, (2) sensational material involving parts of the male or female body or sexual interactions, (3) derogatory comments about well-known figures such as celebrities and politicians, (4) content related to illegal behaviors (eg, fraud or stealing), and (5) false information presented as if it were true. A 5-point response scale was used (0=none, 1=once or twice a year, 2=once or twice in a few months, 3=once or twice a month, and 4=once or twice a week). The sum of the 5 responses was used for the final analysis.

### Victimization

Six items were used to measure whether or not participants experienced cyber violence. The instruction question was as follows: “In the most recent year, have you experienced cyber violence as listed below while you were using the internet/smartphone? If so, how often have you experienced them?” The 6 types of cyber violence outlined were “Someone made derogatory comments about me or hurt my feelings,” “Someone made a false rumor or spread an exaggerated story about me,” “Someone kept sending emails/messages or posting pictures or messages on my blog and social networking site knowing that I do not like,” “Someone sent me sensational pictures or videos without my consent when knowing I do not like,” “Someone posted my personal information (eg, name, address, the name of the school, photos) without my consent,” and “Someone stopped me from exiting the chatroom and made fun of me or made derogatory comments about me and did not let me participate in the conversation.” A 6-point response scale was used (0=none, 1=once or twice a year, 2=once or twice in a few months, 3=once or twice a month, 4=once or twice a week, and 5=almost every day). The sum of the 6-item responses was used to measure victimization.

### Perpetration

Six items were used to measure whether or not participants had been perpetrators of cyber violence. The instruction question was as follows: “In the most recent year, have you done anything that is listed below using the internet/smartphone? If so, how often have you done them?” Six items were asked: “I made derogatory comments or hurt other people’s feelings,” “I made a false rumor or spread an exaggerated story about others,” “I kept sending emails/messages or posting pictures or messages on other people’s blogs and social networking site knowing that other people don’t like it,” “I sent sensational pictures or videos without consent when knowing other people don’t like it,” “I spread the personal information of others (eg, name, address, the name of the school, photos) without a consent,” and “I stopped someone from exiting the chatroom and made fun of them or made derogatory comments and did not let them participate in the conversation.” A 6-point response scale was used (0=none, 1=once or twice a year, 2=once or twice in a few months, 3=once or twice a month, 4=once or twice a week, and 5=almost every day). The sum of the 6-item responses was used to measure perpetration.

### Average Internet Use

Average internet use per day was measured using a 6-point scale (0=less than 1 hour, 1=1-2 hour(s), 2=2-3 hours, 3=3-4 hours, 5=4-5 hours, and 6=5 or more hours).

### Data Analysis Procedure

For data analysis, we conducted first-, second-, and third-order regression analyses to predict factors that are related to the victimization of cyber violence. In the first-order regression analysis, we included age, average internet usage, COVID-19, and gender as predictors of cyber violence victimization. For the second-order regression model, we added exposure to violent media content and the experience of being a perpetrator to the model. As it has been reported that victimization and perpetration are highly correlated [36], we included perpetration in our second model to account for its impact on victimization. For the third-order regression, we included interaction terms with COVID-19 to account for the impact of COVID-19 on cyber violence victimization. To reduce multicollinearity, all the continuous predictors were mean-centered [47].

## Results

### The Incidences of Exposure to Cyber Violence, Victimization, and Perpetration

In terms of exposure to the 5 categories of cyber violence, 1859/9737 (19.09%) participants reported exposure to at least one category. The most common category in each year was violent content, with 2356/4779 (49.30%) of participants in 2019 and 2573/4958 (51.90%) in 2020 reporting that they had seen violent content at least once or twice per year. Additionally, 329/4779 (6.88%) of participants in 2019 and 436/4958 (8.79%) of participants in 2020 indicated that they had been exposed to violent content once or twice per week. In total, 3192/4779 (66.79%) of 2019 participants and 3326/4958 (67.08%) of 2020 participants reported that they had been exposed to violent media content at least once or twice per year.

Regarding victimization, 903/4779 (18.90%) of students in 2019 and 956/4958 (19.28%) of students in 2020 reported that they had experienced cyber violence at least once or twice per year. Furthermore, 855/4779 (17.89%) of 2019 students and 461/4958 (9.30%) of 2020 students reported that they had been perpetrators of cyber violence at least once or twice per year.

### Hierarchical Moderated Regression Models

The hypotheses were examined with a hierarchical moderated regression. Tables 1-3 present the regression results.

**Table 1:** First-order regression analysis on victimization (N=9737).^a^

Analysis	B	SE	β	*t*	Significance
Constant	6.89	0.04	N/A^b^	194.08	<.001
Age	–0.04	0.01	–.06	–6.09	<.001
Average internet use per day	0.16	0.01	.18	13.83	<.001
COVID-19 (2019=0; 2020=1)	–0.30	0.04	–.10	–0.77	<.001
Gender (male=0; female=1)	–0.12	0.03	–.04	–3.90	<.001

^a^*F*_4,9732_=53.75, adjusted *R*^2^=0.02, *P*<.001.

^b^N/A: not applicable.

**Table 2:** Second-order regression analysis on victimization (N=9737).^a^

Analysis	B	SE	β	*t*	Significance
Constant	6.65	0.31	N/A^b^	211.29	<.001
Age	–0.06	0.01	–.10	–9.65	<.001
Average internet use per day	0.07	0.01	.08	6.80	<.001
COVID-19 (2019=0; 2020=1)	–0.12	0.04	–.04	–3.45	<.001
Gender (male=0; female=1)	–0.01	0.03	–.00	–0.32	.75
Exposure to violent content	0.05	0.00	.14	13.89	<.001
Experience of being a perpetrator	0.57	0.01	.44	48.36	<.001

^a^*F*_2,9730(change)_=1448.08, *R*^2^_change_=.22, *P*<.001.

^b^N/A: not applicable.

**Table 3:** Third-order regression analysis on victimization (N=9737).^a^

Analysis	B	SE	β	*t*	Significance
Constant	6.66	0.04	N/A^b^	163.36	<.001
Age	–0.04	0.01	–.07	–4.93	<.001
Average internet use per day	0.05	0.02	.06	3.68	<.001
COVID-19 (2019=0; 2020=1)	–0.07	0.05	–.02	–1.42	.16
Gender (male=0; female=1)	0.03	0.04	.01	0.87	.38
Exposure to violent content	0.04	0.01	.12	8.31	<.001
Experience of being a perpetrator	0.46	0.01	.36	32.19	<.001
COVID-19 × age	–0.03	0.01	–.04	–2.675	.01
COVID-19 × average internet use	0.03	0.02	.02	1.41	.16
COVID-19 × gender	–0.07	0.06	–.02	–1.32	.19
COVID-19 × exposure to violent content	0.01	0.01	.03	1.74	.08
COVID-19 × experience of being a perpetrator	0.23	0.03	.10	7.76	<.001
COVID-19 × exposure to violent content × experience of being a perpetrator	0.02	0.00	.06	5.54	<.001

^a^*F*_6,9724(change)_=40.32, *R*^2^_change_=0.02, *P*<.001.

^b^N/A: not applicable.

H2 predicted that the relationship between exposure to violent media and victimization would vary with age, gender, COVID-19, and average internet use. Gender was coded as male=0 and female=1, with male as the reference. COVID-19 was coded as 2019=0 and 2020=1, with 2019 as the reference. To test interaction effects, the dependent variable was regressed onto product terms of independent variables. Simple slope analyses were conducted for significant interaction effects if needed.

The first-order predictors explained a significant amount of variance in victimization: *F*_4,9732_=53.75, adjusted *R*^2^=0.02, *P*<.001. Age (β=–.06, *t*=–6.09, *P*<.001), average internet use (β=.18, *t*=13.83, *P*<.001), COVID-19 (β=–.10, *t*=–7.70, *P*<.001), and gender (β=*–*.04, *t*=–3.90, *P*<.001) were significant. Those who were older experienced less victimization, and those who spent more time online experienced more victimization. These results were consistent with H2, which predicted that the relationship between exposure to violent media and victimization will vary across age, average internet use, COVID-19, and gender. The results are also consistent with H4, which suggested a negative relationship between age and victimization. Males (Y_exp_=6.89) reported significantly more victimization than females (Y_exp_=6.77). Victimization was significantly higher before COVID-19 (Y_exp_=6.89) than after COVID-19 (Y_exp_=6.59). H1 predicted that COVID-19 would affect victimization. The results showed that COVID-19 changed its pattern, supporting H1.

The second-order predictor explained a significant amount of variance in the dependent variable: *F*_2,9730(change)_=1448.08, *R*^2^_change_=0.22, *P*<.001. Exposure to violent media (β=.14, *t*=13.89, *P*<.001) as a newly added predictor was significant. Those who are exposed to violent media experience more victimization. The experience of being a perpetrator was included as a control variable, which significantly predicted victimization (β=.44, *t*=48.36, *P*<.001). In the second-order regression analysis, the impact of gender did not hold as a significant variable explaining victimization (β=.00, *t*=–0.32, *P*=.75). However, age (β=–.10, *t*=–9.65, *P*<.001), average internet use (β=.08, *t*=6.8, *P*<.001), and COVID-19 (β=–.04, *t*=–3.45, *P*<.001) held consistent patterns as the first-order predictor model, significantly explaining victimization. Therefore, H1, H3, and H4 were supported in the second-order regression model.

The third-order predictors explained a significant amount of variance in victimization: *F*_6,9727(change)_=40.32, *R*^2^_change_=0.02, *P*<.001. Results showed that COVID-19 did not moderate the relationship between exposure to violent media and victimization (unstandardized coefficient B=0.01, SE=0.00, *t*=1.74, *P*=.08). Results also showed that COVID-19 did not moderate the relationship between average internet use and victimization (unstandardized coefficient B=0.03, SE=0.02, *t*=1.41, *P*=.16). COVID-19 did not moderate the relationship between gender and victimization (unstandardized coefficient B=–0.07, SE=0.06, *t*=–1.32, *P*=.19). However, COVID-19 did moderate the relationship between age and victimization (unstandardized coefficient B=–0.03, SE=0.01, *t*=–2.67, *P*<.01). In 2019, age was negatively related to victimization (simple slope b=–0.04), but it was more negatively related to victimization in 2020 (simple slope b=–0.07). COVID-19 also moderated the relationship between perpetration and victimization (unstandardized coefficient B=0.23, SE=0.03, *t*=7.76, *P*<.001). In 2019, perpetration was positively related to victimization (simple slope b=0.46), but it was more positively related to victimization in 2020 (simple slope b=0.69).

Compared with the second-order regression model, the impact of COVID-19 on victimization lost its significance (β=–.02, *t*=–1.42, *P*=.16), while age (β=–.07, *t*=–4.93, *P*<.001), average internet use (β=.06, *t*=3.68, *P*<.001), exposure to violent content (β=.12, *t*=8.31, *P*<.001), and the experience of being a perpetrator (β=.36, *t*=32.19, *P*<.001) held their patterns regarding the impact on victimization. H1 was only supported in the first- and second-order regression models, whereas H3 and H4 were supported across all 3 models.

Table 4 is a summary table indicating the variables that were significant in the first-, second-, or third-order regression analyses. It illustrates the patterns of significance among different variables in the models.

**Table 4:** Summary table.

Significant variables	First order	Second order	Third order
Age	Significant	Significant	Significant
Average internet use	Significant	Significant	Significant
COVID-19	Significant	Significant	Not significant
Gender	Significant	Not significant	Not significant
Exposure to violent content	N/A^a^	Significant	Significant
Experience of being a perpetrator	N/A	Significant	Significant
COVID-19 × age	N/A	N/A	Significant
COVID-19 × experience of being a perpetrator	N/A	N/A	Significant

^a^N/A: not applicable.

## Discussion

### Principal Findings

In this study, we aimed to investigate the impact of COVID-19 and exposure to violent media content on cyber violence victimization among adolescents using national surveys conducted in South Korea. The findings are as follows: (1) COVID-19 did not exacerbate the likelihood of adolescents being victimized; instead, it was significantly associated with a decrease in the likelihood of victimization; (2) the impact of internet use, gender, and exposure to violent content on cyber violence victimization did not change depending on whether it was before or after COVID-19, but age and being a perpetrator did; (3) young adolescents were more victimized to cyber violence than older adolescents; (4) exposure to violent content led to cyber violence victimization; and (5) the experience of being a perpetrator was correlated with experiencing cyber violence victimization.

In accordance with prior research on cyber violence, it is conceivable that the heightened internet use [33,34] and exposure to violent content [37,40-42] during COVID-19 might have led many to anticipate a worsening of cyber victimization among adolescents. However, it turns out that COVID-19 did not worsen the likelihood of adolescents being victimized; rather, it was significantly related to the reduction in the likelihood of being victimized. This finding is consistent with the research by Garthe et al [11], who reported a decrease in cyber victimization during the COVID-19 pandemic when comparing data collected from US middle school students from 2019 to 2021. Our study confirms a similar pattern across an even broader age range among South Korean adolescents.

Furthermore, in our third model, we tested various interaction terms with the time factor to compare the impact of COVID-19 on victimization. The explained variance only increased by 2% in the third model, and most interaction terms were not significant. In other words, in terms of internet use, gender, and exposure to violent content, the results did not change depending on whether it was before or after COVID-19. The only significant changes were regarding age and the experience of being a perpetrator.

The findings have implications on how we should view the impact of COVID-19 on cyber violence. Many have speculated that COVID-19 would lead to heightened rates of cyber violence among adolescents, attributing this assumption to the natural increase in time spent online and heightened exposure to various types of content. This argument is strengthened by the fact that some studies have identified a significant increase in abusive content generated on social media platforms during the pandemic [48]. However, it turns out that COVID-19 did not intensify cyber victimization; rather it decreased during the pandemic, at least in South Korea. The study shows that we need to be aware of how we should interpret the impact of COVID-19 and that we need to carefully examine other factors that may have contributed to the decrease in cyber victimization.

One possible explanation is that cyber victimization may be closely related to offline victimization. Because of the stay-home measures, most schools had to implement remote learning during COVID-19, which may have helped break the vicious cycle of offline bullying and victimization among adolescents. Previous studies suggest that perpetration and victimization in cyberbullying are related to interpersonal peer aggression [14,36]. For instance, two-thirds of the adolescents who have reported being exposed to cyberbullying in the previous month mentioned that they were also bullied in person at school during the same period [35]. Similarly, over three-quarters of cyberbullying perpetrators admitted that they also bullied others in person within 1 month [35]. These findings indicate the possibility of offline violence continuing in cyberspace as a form of cyber violence. In that sense, COVID-19 may have prevented individuals from being exposed to other in-person violence among peers, and that may have led to less victimization online. This argument has been raised in a previous study addressing the role of face-to-face interactions and how this may fuel online bullying [11].

Reduced cyber victimization can also be explained by the quality of social interaction made online. Previous studies have indicated that increased smartphone use and online communication build social capital, leading to greater psychological well-being among adolescents [39]. This underscores the importance of considering the types of people adolescents interact with, the online communities they are exposed to, and the content they encounter, rather than solely focusing on the total time spent online, when evaluating cyber violence victimization. Marciano et al [9] found a positive correlation between ill health and social media use, and between ill health and media addiction, during COVID-19; however, they emphasized that not all forms of digital media use are detrimental to one’s mental health. Specifically, engaging in one-on-one communication within online friendships in the midst of the pandemic alleviated feelings of loneliness and stress. This demonstrates that the use of media technology is relevant to the mental health of adolescents and to their exposure to cyber violence victimization.

As both the previous literature and this study suggest the potential for interpersonal violence to translate into cyber violence, future studies need to examine the intricate connections between offline and online violence, as well as how violence crosses over between these 2 spaces. In addition, future research should place a greater emphasis on the agency of adolescents, particularly by examining the types of media that adolescents choose to engage with and the communities to which they are most exposed. A comprehensive understanding of the context of the online environment, as well as the proclivities of individuals navigating specific online spaces, will provide valuable insights that can guide adolescents in minimizing cyber violence victimization.

While interventions directly suggested by our findings are beyond the scope of this study, future researchers should consider creative approaches to intervening in media use among adolescents. Rather than focusing on regulating internet usage, we may have to focus more on real-life interventions so that adolescents are not exposed to cyber violence that stems from in-person violence at school.

According to our data, younger adolescents are more likely to be victimized by cyber violence; therefore, it may be worthwhile to intervene earlier rather than later in adolescence to reduce cyber victimization. Early education on cyber violence can prevent both victimization and perpetration. If we manage to lower perpetration, it also has a significant impact on victimization because being a perpetrator and being exposed to cyberbullying are correlated [37]. Exposure to cyber victimization is also associated with exposure to violent content. Paying attention to what type of media content early adolescents are exposed to is crucial because it impacts not only what they see online but also what kind of online communities they build.

### Limitations and Future Research

This study does not nuance the specific types of media content to which adolescents were exposed, nor does it address how offline and online social systems may contribute to the content that adolescents encounter. Maghsoudi et al [49] proposed that online social capital moderates the relationship between online risk exposure and psychological distress. Their study suggested that adolescents who heavily rely on online social capital may be more vulnerable to risk exposure than those who rely less on online social capital. Hence, it is critically important to establish offline support systems for adolescents who are exposed to violent content or cyber violence. Emphasizing online literacy education is also vital for empowering adolescents to cultivate positive online social capital and avoid communities that may pose a higher risk of cyber victimization. Exploring these areas could be valuable for future research. Another limitation of the study is the difference in survey distribution modalities between 2019 and 2020 due to the impact of the COVID-19 pandemic. In 2019, the survey of adolescents was conducted on-site in a classroom setting. However, the 2020 survey was conducted entirely online due to the restrictions of COVID-19. While some research has shown minimal significant discrepancies in results between offline and online surveys, our study was not able to account for the potential differences in results that could stem from the differences in these survey methods. Caution should also be exercised in interpreting the results, as the data are second-hand and based on a self-report survey. Although questions continue to be raised about the veracity of information from self-report methods [50], self-report data are neither inherently valid nor invalid; rather, they vary with the methodological sophistication of the data collection [51]. The validity of self-report information can be improved by minimal standardization of the procedures and high motivation and clear cognitive processes of the participants [51]. As the survey has been conducted annually since 2013, and focus groups of students and teachers have been studied regarding the questionnaire items and the procedures [52], the minimum validity of the data would be guaranteed.

In this study, we examined cyber violence data from a national survey conducted in South Korea, limited to the years 2019 and 2020. Our findings are specific to these 2 years, and we recognize that the trends may differ because COVID-19 would presumably have long-term effects. At the same time, adolescents have become more accustomed to remote interactions since the advent of COVID-19. For future research, we intend to conduct longitudinal data analyses and examine whether the trends in victimization have remained consistent.

### Conclusions

This study sheds light on the complex relationship between COVID-19, exposure to violent media content, and cyber violence victimization among adolescents. The unexpected finding that COVID-19 was associated with a decrease rather than an increase in cyber victimization, or that it had no significance in cyber victimization, challenges common assumptions and underscores the need for nuanced approaches to understanding and addressing cyber violence. Furthermore, the study raises the possibility of the interconnectedness of offline and online violence, suggesting that interventions targeting cyber victimization should consider broader social contexts. Moving forward, policy makers, educators, and researchers must collaborate to develop comprehensive strategies that address both offline and online forms of violence, promote positive online interactions, and empower adolescents with the necessary skills to navigate digital spaces safely. By doing so, we can work toward cultivating a safer and more inclusive online environment for adolescents worldwide.

## Abbreviations

KCC: Korea Communications Commission
PTSD: posttraumatic stress disorder

## Data Availability

The data sets analyzed for this study will be available from the corresponding author upon reasonable request.
